# 

**Published:** 1889-03-30

**Authors:** 


### CONTENTS.

**London to be Ambulanced** - 403

**The Breakdown of the Hospital System.** By LAWSON TAIT, F.R.C.S. - 404

**The Doctor's Pulpit.**—Mental Excitement - 405

**The Court and the Hospitals** - 406

**The Watcher** - 406

**Military Graveyards and Epitaphs in India**—V. By an OLD INDIAN - 407

**Within the Wards.**—Science and Surgery—The Skin as an Indicator - 409

**Notes and News** - 410

**Everybody's Page** - 412

**The Scandal at St. John's H spit l for the Skin** - 412

**Annotations.**—Common Humanity in Lancashire—Fancied Pains—Mr. Gladstone's Constituents in Debate - 413

**Hints for the Sick Room.** By M. M. BASIL, M.A., M.B., etc.—The Importance of Posture - 414

**False Impressions.** By CAROLINE FOTHERGILL, Author of "An Enthusiast," "A Voice in the Wilderness," etc. - 415

**Scraps and Gleanings—Amusements and Relaxation** 418

**EXTRA SUPPLEMENT—THE NURSING MIRROR.**

**En Passant.**—A Nurses' Savings Fund—Asylum Attendants—What Nurses should Know—A Club-room for Nurses—The Southampton Institute—Worcester City Institution—Combination of Private Nurses—Heroism - ci

**Nurses on their Travels.**—Another Letter from Madeira - cii

**The Little One's Mite** - cii

**Everybody's Opinion.**—Nursing the Insane—The Care of the Insane - cii

**Appointments—Vacancies** - civ

**Notes and Queries** - civ

